# A296 ENDOSCOPIC RETROGRADE CHOLANGIOPANCREATOGRAPHY AND PRIMARY SCLEROSING CHOLANGITIS: A RETROSPECTIVE STUDY OF A HIGH-VOLUME PROGRAMME

**DOI:** 10.1093/jcag/gwad061.296

**Published:** 2024-02-14

**Authors:** K Leung, M Youssef, Y Xiao, C Streutker, N Calo, A Gulamhusein, B Hansen, G May, J Mosko, G Hirschfield

**Affiliations:** Toronto Centre for Liver Disease Francis Family Liver Clinic, Toronto, ON, Canada; University of Toronto Temerty Faculty of Medicine, Toronto, ON, Canada; University of Toronto Temerty Faculty of Medicine, Toronto, ON, Canada; Unity Health Toronto, Toronto, ON, Canada; Unity Health Toronto, Toronto, ON, Canada; Toronto Centre for Liver Disease Francis Family Liver Clinic, Toronto, ON, Canada; Erasmus MC, Rotterdam, Zuid-Holland, Netherlands; Unity Health Toronto, Toronto, ON, Canada; Unity Health Toronto, Toronto, ON, Canada; Toronto Centre for Liver Disease Francis Family Liver Clinic, Toronto, ON, Canada

## Abstract

**Background:**

Endoscopic retrograde cholangiopancreatography (ERCP) is a crucial component of care in primary sclerosing cholangitis (PSC).

**Aims:**

In this retrospective study, we sought to characterize the clinical and procedural characteristics of PSC patients undergoing ERCP in a high-volume therapeutic endoscopy centre.

**Methods:**

Using procedural codes and chart review from April 2011 to July 2021, PSC patients who underwent ERCP at St. Michael’s Hospital (SMH) were identified. Chart review of documentation was done to collect clinical attributes, procedural characteristics, pathology, and post-ERCP complications within 90 days. Descriptive statistics and regression analyses were conducted.

**Results:**

There were 167 PSC patients included (69% male, 66% concurrent inflammatory bowel disease). Of 464 ERCPs performed, 64% were patients’ 2^nd^ or more ERCP at SMH. When evaluating patient characteristics per ERCP procedure, median age was 45 yrs. (IQR 31-59), median duration of PSC diagnosis was 6.7 yrs (IQR 2.2-11.0), with cirrhosis diagnosed in 42%. Symptoms at ERCP were jaundice (56%), abdominal pain (43%), subjective fevers (30%) and pruritus (25%). Bloods pre-ERCP demonstrated median ALP 362 U/L (IQR 231-552) and median total bilirubin 76 µmol/L (IQR 29-141). Procedural indications were biliary obstruction (82%), cholangitis (31%) and concern for malignancy (22%). Procedural details are summarized in Figure 1. Stent insertion was associated with concern for malignancy (OR 2.87, 95%CI 1.81-4.55), previous stent insertion (OR 1.73, 95%CI 1.16-2.62) and elevated bilirubin (per unit increase in log[bilirubin] OR 1.53, 95%CI 1.23-1.92). Neoplastic pathology was noted in 20% of satisfactory cytology samples and 27% of biliary/bile duct biopsies, while 86% and 76% had reactive/inflammatory/atypical cells respectively. Post-ERCP complications within 90 days (ascertained in 423 ERCPs) was reported in one-third (127, 31%). The most common complications were biliary blockage (23%), post-ERCP cholangitis (15%), and stent failure (9.3%). Stent insertion was significantly associated with 90-day post-ERCP complications on multivariable analyses accounting for per patient clustering, sex, age, relevant stricture presence, stone removal, target location, symptoms, and pre-ERCP bilirubin level.

**Conclusions:**

Patients with PSC who undergo ERCP have high disease burden. Stent insertion is associated with a sicker PSC phenotype with more obstruction (i.e., higher bilirubin, concern for malignancy); this may explain its association with higher 90-day post-ERCP complications. As this study is limited in its single centre retrospective nature, large-scale administrative data studies are needed to characterize and define optimal use of ERCP in the management of PSC.

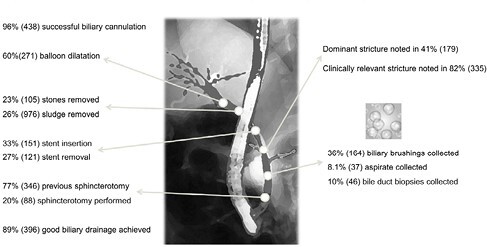

**Figure 1.** Procedural characteristics of 464 endoscopic retrograde cholangiopancreatographies in 167 patients with primary sclerosing cholangitis.

**Funding Agencies:**

None

